# Towards the automatic detection of social biomarkers in autism spectrum disorder: introducing the simulated interaction task (SIT)

**DOI:** 10.1038/s41746-020-0227-5

**Published:** 2020-02-28

**Authors:** Hanna Drimalla, Tobias Scheffer, Niels Landwehr, Irina Baskow, Stefan Roepke, Behnoush Behnia, Isabel Dziobek

**Affiliations:** 1 Department of Psychology, Humboldt-Universität zu Berlin, Unter den Linden 6, 10099 Berlin, Germany; 2 Berlin School of Mind and Brain, Humboldt-Universität zu Berlin, Unter den Linden 6, 10099 Berlin, Germany; 3 Digital Health Center, Hasso Plattner Institute, University of Potsdam, Prof.-Dr.-Helmert-Str. 2-3, 14482 Potsdam, Germany; 4 Institute of Computer Science, University of Potsdam, Am Neuen Palais 10, 14469 Potsdam, Germany; 5 Leibniz Institute for Agricultural Engineering and Bioeconomy, Max-Eyth-Allee 100, 14469 Potsdam, Germany; 6 Department of Psychiatry, Charité-Universitätsmedizin Berlin, Campus Benjamin Franklin, Hindenburgdamm 30, 12203 Berlin, Germany

**Keywords:** Diagnosis, Signs and symptoms, Autism spectrum disorders

## Abstract

Social interaction deficits are evident in many psychiatric conditions and specifically in autism spectrum disorder (ASD), but hard to assess objectively. We present a digital tool to automatically quantify biomarkers of social interaction deficits: the simulated interaction task (SIT), which entails a standardized 7-min simulated dialog via video and the automated analysis of facial expressions, gaze behavior, and voice characteristics. In a study with 37 adults with ASD without intellectual disability and 43 healthy controls, we show the potential of the tool as a diagnostic instrument and for better description of ASD-associated social phenotypes. Using machine-learning tools, we detected individuals with ASD with an accuracy of 73%, sensitivity of 67%, and specificity of 79%, based on their facial expressions and vocal characteristics alone. Especially reduced social smiling and facial mimicry as well as a higher voice fundamental frequency and harmony-to-noise-ratio were characteristic for individuals with ASD. The time-effective and cost-effective computer-based analysis outperformed a majority vote and performed equal to clinical expert ratings.

## Introduction

Social interaction deficits, which encompass non-verbal communication behavior, such as reading and signaling emotions, are prevalent in many psychiatric disorders.^1^ Difficulties in the encoding of emotions are a defining characteristic of autism spectrum disorder (ASD),^2,3^ but have also been reported for other psychiatric disorders.^4–7^ Similarly, differences in signaling emotions are characteristic for ASD^8^ and common in many other mental disorders.^9^

When diagnosing and monitoring the course of a mental disorder, it is thus crucial to assess a patient’s respective deficits with reliable and time-efficient methods. For an objective description and diagnosis of patients’ deficits in general cognition, standardized tests are available, e.g., the Montreal Cognitive Assessment^10^ or the Mini Mental State Examination.^11^ In contrast, quantifying social interaction deficits is still in its infancy. Apart from a few exceptions, e.g., Magdeburg Test of Social Intelligence^12^, clinicians currently rely on the so-called “clinical gaze”, the implicit knowledge based on experience, which is demanded to be used in some clinical investigations such as the autism diagnostic observation schedule (ADOS).^13^ Practitioners need many years of training to acquire the necessary expertise, which is also difficult to verbalize, teach, quantify, standardize, and validate.

The need for precise and standardized tools to measure social interaction deficits is especially evident in ASD, as deficits in social communication and interaction are the core symptomatology in ASD.^14^ The Autism Diagnostic Interview-Revised (ADI-R) and ADOS represent the gold standard of ASD diagnostics and have proven objective and reliable.^13,15^ Such clinical measures include evaluating the patient’s facial expressivity and gaze behavior by a trained clinician following a standardized protocol. However, interrater-variability may account for inconsistencies regarding diagnostic accuracy.^16^ The lack of feasible standardized diagnostic instruments contributes to a high number of non- or late diagnosed individuals.^17^ Especially individuals with ASD and average or above average intelligence are often diagnosed later in life,^18^ as they develop strategies to compensate for their deficits, which has been referred to as camouflaging.^19^ Thus, ASD diagnostics would greatly benefit from automatic methods measuring social interaction deficits validly and reliably.

Digital standardized tests that automatically analyze a patient’s social behavior would provide a widely accessible time-efficient and cost-efficient alternative to expert diagnosis. Some studies have recently shown the potential of analyzing social behavior, such as speech,^20^ facial expressions,^21^ and gaze behavior^22^ with machine-learning methods to assess general mental disorder status. However, to date paradigms to measure social behavior in a standardized way, in which an interaction partner reacts in a reproducible and comparable way to each participant, are lacking. Consequently, we designed the simulated interaction test (SIT), entailing a short standardized simulated dialog between a participant and an actress about food preferences and dinner preparation.

The SIT evokes aspects of social interaction behavior previously described as atypical for individuals with ASD: Individuals with ASD share emotions of others less intensely, which has been reported for negative^23^ and positive emotions.^24^ The mimicry of facial expressions has been found reduced,^25^ an effect that scales with severity of social dysfunction in ASD.^26^ Atypical gaze patterns^27,28^ are furthermore characteristic of ASD—especially the avoidance of direct eye contact.^29–31^ Moreover, ASD involves aberrant voice intonation,^32^ especially in naturalistic settings.^33^ The SIT aims at reliably capturing those social biomarkers, which may aid earlier diagnosis as well as monitoring of the course of the disorder and treatment outcome.

Recent attempts towards meaningful diagnostic predictions have shown the potential of machine-learning approaches to analyze non-verbal behavior differences to detect ASD.^34–38^ However, to the best of our knowledge, no existing approach focuses on adults with ASD and normal intelligence levels despite the high need for access to diagnosis.^39,40^ Thus, using computer vision and machine learning on video- and audio recordings, in the current study we aimed to identify social biomarkers of ASD that allow an affordable, accessible, and time-effective identification of the diagnosis.

This paper encompasses two studies: In a facial electromyography (EMG) preparatory study, we assessed facial behavior via the SIT in a sample of healthy controls (HC; i.e., individuals without autism). Making use of the high precision of EMG, we aimed at the precise description of non-verbal facial behavior in the simulated interaction to select relevant features (i.e., regions of interest) for the main study. In the ASD study, we aimed to replicate the results for HC using automated methods and compared non-verbal social communication behavior of individuals with and without ASD. Further, we followed up on previous research^41^ predicting the diagnosis of ASD individuals based on interaction behavior, comparatively evaluating the SIT’s diagnostic properties and gold standard clinical measures as well as judgments of clinical experts.

## Results

### Participants

We analyzed the video- and audio-recordings of 120 participants in total, which took the SIT, 80 (ASD: 37, NT: 43) in the clinical main study and 40 male HC in the EMG preparatory study (see Supplementary Information). In the main study, all participants’ video recordings were analyzed by clinical experts as well as by computer-based tools.

### Computer-based analysis

The results of the computer-based analysis showed that individuals with ASD could be detected with varying accuracy based on facial expressions, gaze behavior, or vocal characteristics. For transparent and explainable diagnostic decisions, we limited ourselves strictly to features based on domain-knowledge or on the EMG preparatory study (see Supplementary Information). Due to the strong skewness and non-normality of the data, we only used non-parametric tests.

Nearly all frames (99% in both groups) of the video recording data could be analyzed successfully with the computer vision tool OpenFace^42^ with high confidence (on a scale of 0–1: mean = 0.99 in both groups, std = 0.013). There was no evidence that one of the two groups (ASD, NT) could be tracked with higher confidence (*p* = 0.97). We excluded all frames that were either not tracked successfully or with confidence below 0.75. For a fair comparison of the classifiers, we had to exclude one female participant with autism because of missing audio recording.

First, we investigated facial expressions across groups and examined whether participants expressed the relevant action unit (AU) for the part of the communication involving positive emotions (AU12 and AU6) and negative emotions (AU4). To account for participants’ baseline facial expression we compared mean occurrence and intensity of the AUs relevant for each part to the activity during the neutral part of the communication.

In line with the results of the EMG study, participants showed more positive facial expressions during the positive part of the conversation (talking and listening about favorite dishes) compared to the neural/negative parts. The positive expressions were evident in a higher mean intensity of AU12 (Mdn = 0.30) as well as of AU6 (Mdn = 0.43) in comparison to their intensity in the neutral part, AU12 (Mdn = 0.24; *Z* = *703*, *p* < 0.001) and AU6 (Mdn = 0.33), *Z* = 567, *p* < 0.001). Likewise, the participants showed higher occurrence of AU12 (positive: Mdn = 0.20 vs. neutral: Mdn = 0.10; *Z* = 499.0, *p* < 0.001) and AU6 (positive: Mdn = 0.04 vs. neutral: Mdn = 0.001; *Z* = 232.0, *p* < 0.001) compared to the neutral part.

In line with the preparatory study, participants tended to show more negative expressions during the negative part of the conversation (talking about disliked food), which was evident as a trend towards a higher intensity of AU4 (Mdn = 0.058) compared to the neutral part of the conversation (Mdn = 0.056), *Z* = 1044.0, *p* = 0.06.

To compare the facial expressions of both groups, we focused first on the two emotional parts of the conversation and the respective AUs. In the positive part of the conversation (favorite dish), individuals with ASD (Mdn = 0.12) expressed AU12 (smiling mouth) compared to HC (Mdn = 0.40) significantly less frequently (*U* = 578.0, *p* = 0.018, *z* = 0.27). Regarding AU6 (smiling eyes), we found no evidence for group differences in intensity or occurrence (all *p* > 0.05) in the positive part of the conversation.

In the negative part, individuals with ASD showed more negative facial expressions than individuals without ASD, i.e., more intense AU4 activity (ASD: Mdn = 0.08 vs. NT: Mdn = 0.03), *U* = 568, *p* = 0.014, *r* = 0.29.

Next, we compared social smiling in both groups during the entire conversation. For both relevant AUs (AU12 and AU6), we inspected occurrence and intensity. Individuals with ASD showed less social smiling in the mouth region (AU12) compared to individuals without ASD; this was evident in AUs’ occurrence (ASD: Mdn = 0.09 vs. NT: Mdn = 0.40; *U* = 557.0, *p* = 0.01, *r* = 0.30) as well as intensity (ASD: Mdn = 0.16 vs. NT: Mdn = 0.52; *U* = 581.0, *p* = 0.019, *r* = 0.27). Additionally, individuals with ASD showed less social smiling around their eyes (AU6) compared to individuals without ASD, again evident in occurrence (ASD: Mdn = 0.02 vs. NT: Mdn = 0.12; *U* *=* 598.5, *p* = 0.028, *r* = 0.25).

To measure facial mimicking behavior of the participants, we calculated the time-shifted-correlation of the actress’ facial behavior and the participants’ facial expression behavior. Mimicking the actress’ AU6 activity was more pronounced in HC. This was evident in a higher correlation of the actress’ and participants’ time series in this AU’s activity in the HC group (ASD: Mdn = 0.08 vs. NT: Mdn = 0.19, *U* = 113.0, *p* = 0.04, *r* = 0.34).

Applying a random forest classifier and all of the facial expression features, we could predict an individual’s diagnosis with an area under the ROC curve of AUC = 0.65.^43^ Using all 17 AU provided by OpenFace, we were able to predict the diagnosis with AUC of 0.74 AUC. An exploratory in-depth analysis revealed that more female individuals (34 out of 39) than male individuals (20 out of 40) could be classified correctly based on facial expressions (*X* = 12.34, *p* < 0.001).

**Fig. 1:**
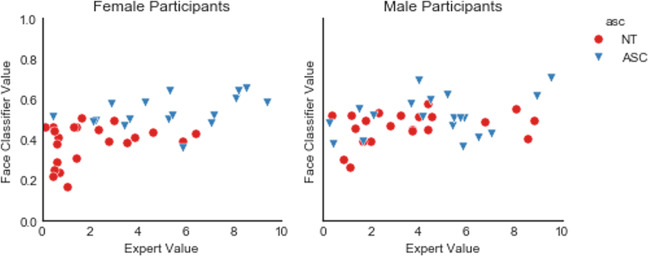
Automated classification based on facial expression separated by gender.

Figure 1 shows the predictions of the face-based-classifier separated by gender and in comparison to experts’ classifications. Adding age as a feature did not improve the accuracy of the classifier.

For gaze analysis, again, we first excluded frames that were tracked non-successfully or with low confidence. Next, we compared the two groups based on gaze variation. We used the individual’s gaze angle in radians in world coordinates, averaged for both eyes, as they are provided by OpenFace.^42^ Three example participants’ raw gaze patterns are displayed in Supplementary Fig. 2.

We compared the groups regarding the following features: difference in variance and means on eye movements of the vertical and the horizontal axis as well as differences in speed of the eye movements. To account for difference in participants’ height or position, we also calculated the absolute deviation of the values from the median of the eye gaze direction. In general, the variation around the median gaze angle was small. Correcting for multiple comparisons, we found no evidence for group differences in gaze behavior. The descriptive values for both groups are presented in Table 1.

**Table 1:** Gaze behavior separated by groups.

	NT	ASD
Mean (gaze angle horizontal)	0.02 (SD: 0.06)	0.01 (SD: 0.07)
Mean (gaze angle vertical)	−0.25 (SD: 0.12)	−0.23 (SD: 0.11)
Absolute deviation from median gaze angle (horizontal)	0.02 (SD: 0.01)	0.03 (SD: 0.01)
Absolute deviation from median gaze angle (vertical)	0.03 (SD: 0.01)	0.033 (SD: 0.01)
Mean speed of eye movement (horizontal)	0.65 (SD: 0.20)	0.67 (SD: 0.21)
Mean speed of eye movement (vertical)	0.71 (SD: 0.32)	0.64 (SD: 0.26)
Mean acceleration of eye movement (horizontal)	0.97 (SD: 0.32)	0.99 (SD: 0.32)
Mean acceleration of eye movement (vertical)	0.93 (SD: 0.42)	0.88 (SD: 0.38)

Using a random forest classifier and all gaze behavior features, we could predict an individual’s diagnosis with an AUC = 0.63. The exploratory in-depth analysis of the classification results showed no significant evidence for different accuracy of classifying male and female participants.

The descriptive values of the voice characteristics for both groups are presented in Table 2. As pitch and harmony-noise-ratio (HNR) vary strongly between males and females,^44,45^ we included participants’ gender in the analysis. A significant main effect of the group supported the assumption of a different fundamental frequency (*F*_0_) in individuals with and without ASD spectrum condition, *F*(1, 76) = 15.51, *p* = 0.0002, *η*^2^_G_ = 0.024. As expected, there was an additional main effect of gender, reflecting the on average higher pitched voices of women, *F*(1, 76) = 559.73, *p* < 0.0001, *η*^2^_G_ = 0.86.

**Table 2:** Voice characteristics including harmony-noise-ratio (HNR) and fundamental frequency (*F*_0_) in Hertz (Hz) for both groups.

	NT	ASD
Mean *F*_0_ (Hz) female	209.08 (SD: 17.97)	218.91 (SD: 16.16)
Mean *F*_0_ (Hz) male	121.68 (SD: 11.68)	139.67 (SD: 16.22)
Median HNR female	10.32 (SD: 1.47)	6.91 (SD:2.12)
Median HNR male	10.84 (SD:1.42)	8.50 (SD:1.44)

Further analyzing HNR, a main effect of group was observed, *F*(1, 76) = 7.97, *p* = 0.0061, *η*^2^_G_ = 0.055, pointing towards a higher HNR in individuals with ASD. As expected, there was an additional main effect of gender, *F*(1, 76) = 60.78, *p* < 0.0001, *η*^2^_G_ = 0.42.

We found no significant evidence for group differences regarding jitter, shimmer, or energy (all *p* > 0.01, corrected for multiple comparisons).

For the machine-learning approach, we also included Mel-frequency cepstral coefficients (MFCCs) and were able to reach an AUC of 0.77. The exploratory in-depth analysis of the classification results showed no significant evidence for different accuracy of classifying male and female participants.

### Clinical expert-based analysis

Given that misclassification of individuals with ASD by automated analysis of the SIT could be due to the paradigm not capturing enough information, we asked clinical experts in ASD diagnosis to rate the non-verbal behavior of participants from the videos to estimate the informative value of the SIT recordings. The clinical experts correctly recognized most of the HC individuals and more than half of the individuals with ASD. Among the eight psychologist/psychiatrist expert raters the accuracy ranged between 0.56 and 1 with a mean of 0.71. There was no evidence that experts classified more female than male individuals correctly (*X* = 6.00, *p* = 0.11). More experienced raters (months of diagnostic or therapeutic work with individuals with ASD) showed a higher percentage of correct classifications (*r*_s_ = 0.72, *p* = 0.044).

### Comparison of ML-classifier and experts

**Fig. 2:**
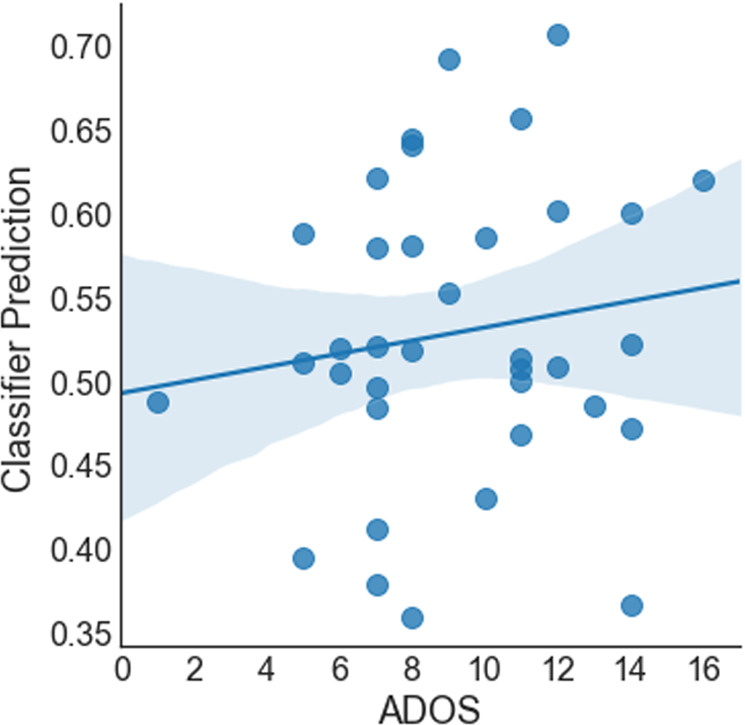
Class probability for ASD and participant’s ADOS score.

**Fig. 3:**
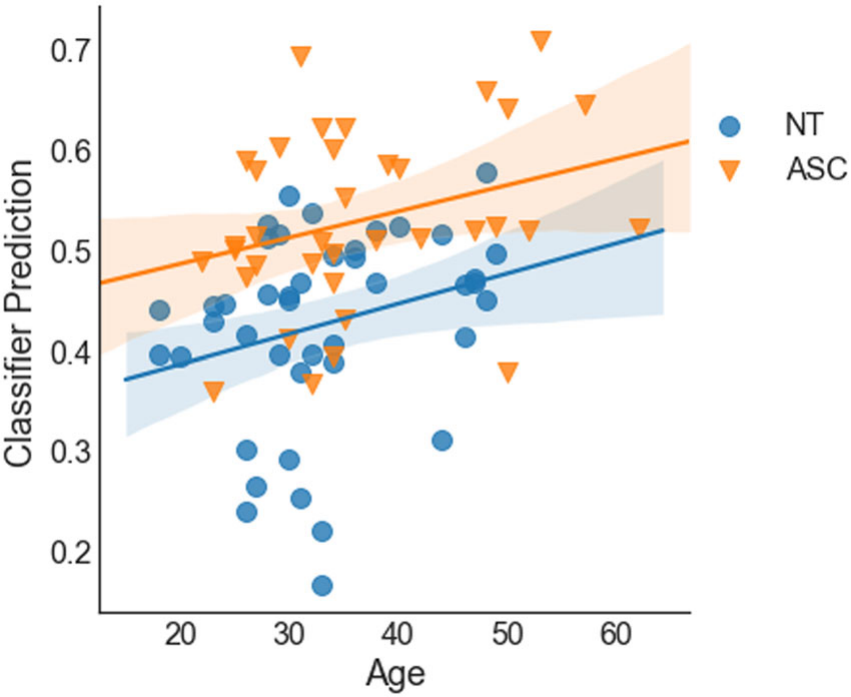
Class probability for ASD and participant’s age.

**Fig. 4:**
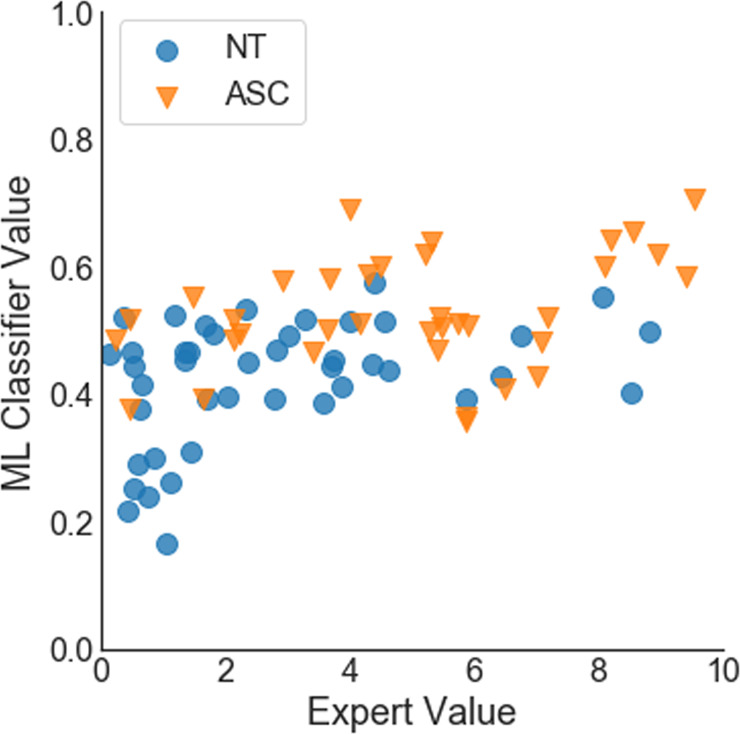
Class probabilities based on machine-learning (ML) classification and expert ratings.

**Fig. 5:**
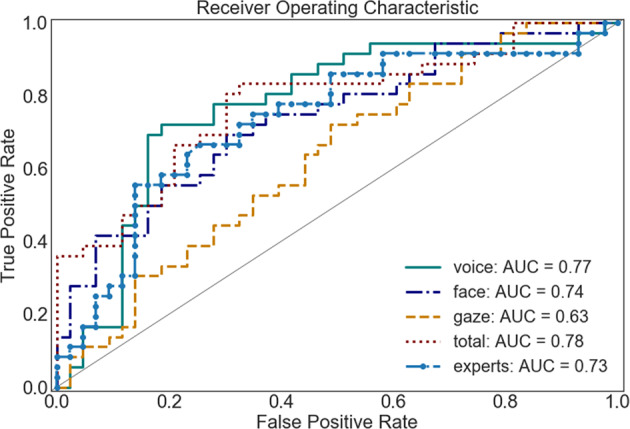
ROC curves of all classifiers.

Combining all features (gaze behavior, voice, and facial expressions), we reached an AUC of 0.78. Setting a threshold at 0.5 class probability, we reached an accuracy of 73%, which was according to McNemar’s Test significantly better than a majority vote (*X* = 9, *p* = 0.014) and not significantly worse than the accuracy of the clinical experts (*p* > 0.05). The sensitivity was 67% and specificity 79%. The detection rate was slightly better for female (33 out of 39) than male (25 out of 40) participants, *X* = 3.88, *p* = 0.048. Further, the class probability for ASD calculated by the classifier scaled positively with participants’ ADOS score (*r*_s_ = 0.48, *p* < 0.0001, see Fig. 2) and with participant’s age (*r*_s_ = 0.35, *p* = 0.001,see Fig. 3). For a comparative overview of the predicted class probabilities based on the machine-learning classification and expert ratings (on a scale of 0–10 and threshold at 5) see Fig. 4. For comparing the ROC curves of all classifiers see Fig. 5. Confusion matrixes of all classifiers are in Supplementary Tables 1–5.

## Discussion

We presented the simulated interaction task (SIT), a cost-efficient and time-efficient digital tool to identify social biomarkers, which we validated in an EMG study in healthy individuals and a clinical study using an automated digital approach in individuals with ASD. To the best of our knowledge, the SIT is the first fully standardized and computer-based measure of social interaction deficits. Using EMG as well as computer-vision-based analyses, we showed that the SIT reliably evoked positive and negative emotional expressions and as such represents a naturalistic paradigm for measuring non-verbal social behavior. Results of the ASD study suggest that the machine-learning-based automated analysis of facial expressions, gaze and voice holds potential to measure social phenotypical behavior and supplement traditional clinical assessment of social interaction deficits.

**Table 3:** Mean standardized activity of each muscle in each conversation part.

	Zygomaticus major (z-stand.)	Corrugator supercilii (z-stand.)	Levator labii (z-stand.)
Dinner preparation (neutral)	−0.21 (SD: 0.86)	0.10 (SD: 1.21)	−0.14 (SD: 0.77)
Liked food (positive)	0.03 (SD:0.94)	−0.05 (SD: 0.83)	0.06 (SD:1.04)
Disliked food (negative)	0.15 (SD:1.22)	−0.04 (SD: 0.92)	0.06 (SD:1.12)

In the preparatory EMG study, participants smiled more when the actress talked about both liked and disliked food, compared to when she explained how she sets a table for dinner. Listening to the actress’s disliked food, they also showed more facial expressions of disgust. Both social smiling and mimicking of joy and disgust replicates literature about typical human behavior in social interactions.^46–48^ In line with the results of the EMG study, participants in the ASD study also expressed more positive facial expressions during the positive part of the conversation and tended to show more negative expressions during the negative part. Thus, the results of both studies validate the SIT as a tool to evoke naturalistic non-verbal social behavior (Table 3).

The preparatory study showed that across the whole conversation, individuals with more pronounced autistic traits expressed less smiling and more frowning. Based on these findings, we selected AUs representing smiling, frowning, and expressions of disgust for the ASD study to analyze their descriptive and diagnostic potential.

Using the SIT in a clinical sample of individuals with ASD, we showed group differences in the expression of facial emotions and voice modulation. Thus, the automated measurement of non-verbal social communication behavior holds potential to complement the phenomenological understanding of ASD. Individuals with ASD expressed less social smiling and less mimicry of positive facial expressions than HC individuals. This is in accordance with previous studies indicating reduced spontaneous mimicry in individuals with ASD.^25,49^ Further, we are replicating the finding of a recent study using computer-based facial expression analysis that reported fewer happy expressions in individuals with ASD.^50^

Further, individuals with ASD spoke on average with higher pitch and higher HNR than NTs. Higher pitch has been described for individuals with ASD before,^51,52^ although some studies reported null-results.^53^ The naturalistic setting of the SIT resembling a common video chat situation might have enabled us to detect these differences more sensitively than previous studies. Interpretation of our HNR result is not straightforward, given the lack of reference studies. One study with children with ASD found a negative association between HNR median and disorder’s severity.^54^ In contrast, a study focusing on adolescents with ASD^55^ found an association of higher median HNR and perceived voice awkwardness. Given this heterogeneity and given that HNR changes with age,^56^ it is difficult to generalize those results to our study sample of adults with ASD. In addition, it cannot be ruled out that individuals with ASD differ from those without ASD regarding their ratios of speaking and pauses, which might have influenced the results. In general, quantitative evidence for voice differences in ASD is lacking, as a recent meta-analysis concluded.^53^ Given the relatively high explanatory power for diagnosis in our study, however, further studies are needed to elucidate the role of HNR in ASD.

We found no significant evidence for group differences regarding gaze behavior, although many studies have reported differences before^57^ and aberrant gaze has recently been discussed as a potential biomarker of ASD.^58^ An explanation for our null-result might lie in the fact that the appearance-based gaze estimation used here, in comparison to eye tracking methods that were used previously, was not sensitive enough to detect the subtle gaze dysfunctions present in ASD. The results of the machine-learning-based analysis described below are in favor of this interpretation.

Comparing the groups regarding single features of socio-emotional behavior can be informative. However, the problem of multiple testing remains, as the automated analysis allows the comparison of many different aspects of behavior. Thus, we see a clear advantage of multi-dimensional approaches like machine learning that allow the capturing of the multivariate and integrated nature of naturalistic social behavior and analyze its diagnostic value.^59^ Further, as we carefully split our data in train and test sets, we received information about the predictive value of the behavioral differences for individuals, which were not part of the training sample for the model.

The automated analysis of the SIT reached an accuracy of 73% at a set threshold of 0.5 class probability. The predicted class probabilities were associated positively with participants’ overall ASD symptom severity. Only few studies using machine learning to detect ASD reached higher accuracies based on, e.g. automatic analysis of upper-limb movements,^34^ or eye movements in a face-recognition task.^35^ However, both studies used small sample sizes and were directed towards more affected children and not high-functioning adults with autism, which present with more subtle deficits. Further, those studies used high-cost apparatus, not suited for large-scale testing. In contrast, the application of the SIT only requires a standard PC, a camera, and a microphone. Thus, individuals can take the SIT at their home computers, which ensures, especially for individuals with autism, a more naturalistic setting than in a laboratory, leading to higher external validity and reducing confounding factors such as anxiety.

Based on facial expressions, more women than men could be classified correctly. One possible explanation might be that women with autism require more severe symptoms to receive an ASD diagnosis.^60^ As our sample includes only individuals with ASD that were already diagnosed, the female individuals with ASD might have been easier to detect automatically based on their more dysfunctional facial expressions than the male participants.

Both classifications on all facial AUs available via OpenFace and on all voice characteristic including MFCC revealed good results pointing to the value of multidimensional representations of phenotypes. The classification based on gaze behavior was remarkably worse than the prediction based on vocal and facial behavior. Post hoc analysis of the gaze’s variance suggests that this is likely due to the low precision of the eye gaze measurement. Also, the null finding of group differences in gaze behavior has to be interpreted with caution, as automated gaze tracking might not be accurate enough for computation of velocity and acceleration. Due to the relevance of gaze behavior in autism spectrum conditions,^31^ further research should follow up on this with using state of the art eye-tracking devices to estimate the information value of gaze within a simulated interaction.

The classification based on the short recording of overall social communication behavior was not significantly worse than the clinical experts in detecting ASD diagnostic status. However, the judgment performance of the expert raters varied considerably from approximately chance level to 100% accuracy and scaled positively with experience. This can be seen as a strong argument for the application of the SIT, which is reliable as well as cost-efficient and time-efficient, while the clinical assessment does not only require intensive training but also, as our data indicates, continuous experience. Nevertheless, it should be highlighted that the SIT is not meant to substitute traditional clinical assessment but rather to allow additional information about the client’s social communication abilities at an early stage of the diagnostic process.

Despite the substantial prevalence of ASD of 1 in 59 children,^61^ there is only a limited amount of practitioners with expertise in diagnosing ASD.^62^ As a result, many individuals, especially with high-functioning ASD, are diagnosed late^18^ or not at all,^63^ resulting in substantial burden.^64^ In contrast to clinical interviews that require trained experts, the SIT offers the potential of a widely accessible and easily applicable screening tool for ASD, even in rural areas with limited access to clinical care. Therefore, the SIT may enable time-efficient detection of social interaction deficits and low-cost screenings of large populations. However, it is important to keep in mind that the SIT was designed as a tool to enrich and standardize the assessment of social communication behavior to supplement clinical diagnosis, screening, and treatment monitoring instead as a stand-alone diagnostic or screening tool. Nevertheless, it is crucial to understand the impact on individuals and health care systems of employing such automated behavior measurement tools and providing feedback based on them. On the one hand, the SIT offers a more standardized, more accessible and cost-effective measurement of non-verbal social communication behavior than clinical interviews and thus could improve screening, diagnostic, or monitoring processes. On the other hand, especially screenings and unsupervised feedback might result in more individuals seeking care and lead to higher costs for the health system. It is warranted to carefully define the area of use and evaluate benefits, costs, and risks.

The importance of monitoring and characterizing social communication behavior is not limited to ASD, but applies to many other psychiatric conditions, as differences in recognizing and signaling emotions are common phenomena in mental disorders and vary with disorder severity, e.g., in psychosis,^4,65^ depression,^5^ bipolar disorder,^6^ and substance use disorders.^7^ Thus, the SIT may serve not only as a diagnostic instrument but also as a tool for measuring treatment-based outcomes in other conditions than ASD. Thus, we believe it is warranted to explore the SIT’s specificity and sensitivity in larger clinical samples with different psychiatric conditions.

Interestingly, older participants in both groups were more often labeled as autistic than young participants by the machine-learning classifiers, as the classifier was probably picking up a tendency of higher age in the ASD group. This age bias points to the strong need of careful testing for confounders and biases in training data for all kind of machine-learning diagnostic methods.

Aiming for a high standardization, we prerecorded the actress’ part of the conversation. Thus, she responds in exactly the same way to each participant at the same time point—independent of participant’s response content or length. That being said, we took several measures to make the conversation flow as naturally as possible. First, the conversation is set up in such a way—and the participant is informed about this during the instructions—that the actress poses a series of questions, which the participant is asked to provide an answer for. Thus, the participant expects those questions rather than tailored replies to her answers. In addition, the participants’ answer sections were made as natural as possible by the actress displaying empathic listening behavior, such as e.g. nodding, which would be appropriate for the kind of small talk conversation at hand. Second, we gave clear instructions (and provided an exercise) to answer in three to four sentences to the actress’ questions: the conversation actually starts with a trial where the actress tells the participant how she got into the institute and asks the participant to describe how she got into the institute in three to four sentences.

However, future versions of the task would benefit from tailored answers of the actress that are adaptive to the length of the interactants’ answers. For this first version of the SIT we aimed at the highest possible standardization though and asked the participants to behave as they would in a real interaction. In accordance, the majority of participants in the EMG-study reported post hoc that they behaved similar to a real conversation when talking to the actress. To further evaluate this aspect in the future, the current version of the SIT ends with three follow-up questions, how naturalistic the participant perceived the interaction. Further measures should be implemented to allow for home setting use (e.g. automated checking of camera and microphone) and research should be carried out to evaluate the accuracy of the SIT in a home setting, as self-administration of tests often leads to diminished accuracy.

Future studies should be undertaken to compare the behavior in the simulated interaction with behavior in a face-to-face control condition. A live setting with the same topic and a dialog script could inform about the validity of the simulated procedure. As similarity enhances facial mimicry,^66^ further version of the SIT could provide different interaction partners matching the participant’s gender, age, or ethnical background.

Taken together, combining standardization with a naturalistic paradigm, the SIT allows to objectively quantify social communication behavior and qualifies as a cost-efficient and time-efficient digital tool to detect social biomarkers in ASD.

## Method

### Preparatory study

In a facial EMG preparatory study (cf. Supplementary Information), we assessed facial movement behavior via the SIT in a sample of healthy male individuals. Making use of the high precision of EMG, we aimed at the precise description of non-verbal facial behavior in the simulated dialog to select relevant features for the main ASD study.

### Participants

Thirty-seven adults with ASD (18 females, mean age = 36.89 years, range = 22–62) and 43 HC individuals (22 females; mean age = 33.14 years, range = 18–49) were enrolled in the study. Individuals with ASD were recruited via the ASD outpatient clinic of the Charité— Universitätsmedizin Berlin. All of the participants with ASD had previously received a diagnosis of Asperger syndrome, atypical ASD or childhood ASD based on ICD-10 criteria.^67^ The diagnostic procedure included the ADOS (*n* = 35^68^) and, for patients with available parental informants, the ADIR (*n* = 21^69^).

Only participants without current antipsychotic and anticonvulsant medication as well as comorbid neurological disorders were included. In addition, we set the maximum age for inclusion at 65 years to avoid possible age-related neurodegeneration. Furthermore, we used a German vocabulary test for verbal intelligence (Wortschatztest)^70^ to ensure sufficient language command. In the control group, any history of psychiatric disorder led to exclusion. Based on these criteria, we excluded three participants.

### Procedure

The study took place in a quiet laboratory with constant lighting conditions. After having given informed consent, participants were asked to sit in front of a computer screen. The participants were informed that they were to have a short conversation with a woman, whose part was recorded before, and were encouraged to behave as they would in a natural conversation. The experimenter then left the room and the participant started the SIT. During the whole simulated interaction, the participant’s face, gaze behavior, and voice were video and audio recorded, respectively, and analyzed later using computer-based technologies. The video of the participant was recorded automatically and timestamped to later align the actress’s and participant’s recordings. Participant’s autistic traits were assessed via the Autism-Spectrum Quotient.^71^ All participants gave written informed consent prior to participation. All individuals displayed in figures have given their consent for publication. The study was approved by the ethics committee of the Charité—Universitätsmedizin Berlin and was conducted in accordance with the Declaration of Helsinki.

### Simulated interaction task

**Fig. 6:**
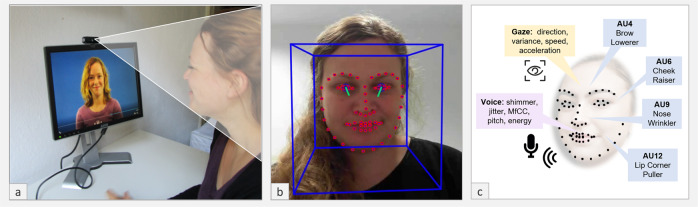
Experimental setting und automated analysis of SIT. **a** Neurotypical participant taking the SIT. **b** Face-tracking and gaze-tracking using OpenFace. **c** Facial landmarks and features of main interest; written informed consent was obtained from the persons to have their photos used in this study.

The SIT (Fig. 6) is a simulated social interaction designed as a conversation between the participant and an actress about food preferences and dinner preparation, where the part of the actress is prerecorded. In the first part of the conversation (3.08 min), the actress introduces herself and asks the participants for their names for a short warm-up. Next, she explains the task and the topics of the following conversation to the participants and, as an example, asks the participant to describe how they got to the institute. This part of the conversation represents a warm-up for the dialog and is not analyzed. Thereafter, the actress introduces the three topics that the conversation will be about, i.e., dinner preparations and liked/disliked food and announces that participants will be given time to think about their answers before the participant starts the task by button press. This preparatory phase ensures the conversation’s flow and thus approximates real-life conversation conditions.

**Fig. 7:**
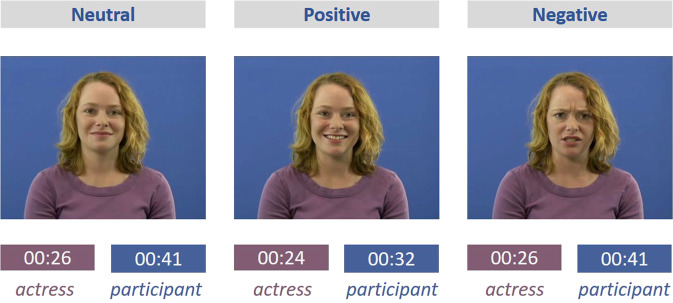
Timing for each excerpt of SIT. Written informed consent was obtained from the person to have her photos used in this study.

In the following recording (3.2 min), the actress addresses the participant in three excerpts about (i) how she usually sets a table for dinner (26 s), (ii) food she likes (24 s), and (iii) food she dislikes (26 s). Following each section, she asks the participant about the same respective preferences and information. After each question, the participant has about half a minute time to answer (see Fig. 7). While the participant answers, the actress displays empathic listening behaviors, e.g., she smiles and nods at the participant. Thus, the SIT allows to objectively measure qualitative and quantitative differences in social communication behavior with a high-level of standardization and in an interactive naturalistic manner.

Recent research in the area of social cognition has emphasized the strong need for more interactive experimental social tasks for basic research and the clinical diagnosis process.^72^ However, interactive paradigms are often more time consuming and financially costly, as they require a second participant, a confederate or professional to interact with the participant. Standardization is furthermore a challenge as the participant’s counterpart rarely interacts in the exact same manner. In contrast, the SIT uses a video conversation setting, which today represents a familiar interaction setting for many individuals, as they are common both in private and business context. Using a prerecorded partner, the SIT does not demand a second person for testing. We chose food preferences as conversation topic for the SIT, as this represents a typical small talk subject that allows for the elicitation of positive and negative emotions in a short amount of time without involving highly personal or sensitive experiences.^73^ Moreover and importantly, conversation excerpts vary considerably less in length than would be the case with other topics that have been used in experimental settings (e.g., favorite films) which thus facilitates standardization. Given those considerations, the SIT represents a quasi-naturalistic setting, balancing free interaction and standardization.

During the shooting of the video, in order to generate natural dialog behavior, the actress actually conversed with the film director about food preferences, which thus not only involved her talking about her preferences but also listening to his. The actress was instructed to show empathic communication behavior, entailing generally positive attitude towards the interaction partner, including confirmative nodding und social smiling. The duration of these parts for the SIT was estimated based on time needed from sample participants.

The SIT allows a non-intrusive measurement of non-verbal communication behavior, which demands a computer with camera and microphone only and can thus be conducted in a laboratory and home setting. In particular clinical studies may benefit from the non-intrusive nature of the task, as additional equipment such as EMG electrodes might hinder naturalistic behavior and introduce other confounds (e.g. aversive reactions in touch-sensitive ASD patients).

We validated and quantified the actress’ non-verbal behavior by analyzing her video with automatic facial expression analysis, focusing on AUs most relevant for social behavior and the specific emotions addressed (AU6, AU12, AU9, AU4). The actress shows more expression of disgust (AU4: *M* = 0.23, AU9: *M* = 0.07) and less expression of joy (AU6: *M* = 0.61, AU12: *M* = 0.72) when she speaks about disgusting food versus speaking about her favorite food (AU4: *M* = 0.06, AU9: *M* = 0.05, AU6: *M* = 0.97, AU12: *M* = 1.2).

The same pattern, indicating mimicry behavior, is evident when she listens to the participant talking about their favorite (AU4: *M* = 0.02, AU9: *M* = 0.04, AU6: *M* = 1.15, AU12: *M* = 1.36,) versus non-favorite food (AU4: M = 0.13, AU9: M = 0.06, AU6: *M* = 0.95, AU12: *M* = 1.11).

As expected for empathic listening and representing affiliative behavior, a comparison of the actress listening versus talking revealed that the actress smiles more when she listens (AU6: *M* = 0.53; AU12: *M* = 0.92) than when she speaks (AU6: *M* = 0.33, AU12: *M* = 0.79).

### Automatic data analysis

We analyzed face, gaze, and voice recordings using the open source toolkit OpenFace^42^ and python library Librosa^74^ and Parselmouth Praat Scripts in Python by David Feinberg.^75^ If not otherwise specifiecd, all statistical tests are two-sided. Based on the literature and the EMG preparatory study, we focused on features representing typical social and affiliative behavior and explain them in more detail in the following sections:

OpenFace identifies small observable movement in facial AUs using computer-based analysis. We followed the same data analysis procedure for this automatic approach as for the EMG data. First, we excluded every participant who was not tracked successfully in more than 90% of the frames or with a mean confidence below 0.75. The frame confidence is computed by OpenFace via training a separate confidence network that is trained to predict the expected landmark detection error. Second, we measured whether facial expressions matched the predefined emotion for each conversation part (e.g., happiness in the excerpts dealing with positive food preferences). To this end the intensity of the corresponding emotional facial expression was measured, operationalized as the mean activity of the relevant AUs based on Ekman and Friesen,^76^ i.e., for joy AU6 (Orbicularis Occuli) and AU12 (Zygomaticus Major) and for disgust AU9 (Levator Labbi) and AU4 (Corrugator Supercilli). The neutral part was used as a baseline measurement for the other two parts.

Further, we assessed the participant’s mimicking of the actress’ facial expressions: For each relevant AU, we calculated a correlation between the actress’ and the participant’s activity within 10-s timeframes. Tracking affiliative behavior and based on the finding of the EMG preparatory study, we further measured the individual’s social smiling during the entire conversation (AU12 and AU6).

Before analyzing the gaze behavior, we excluded frames tracked unsuccessfully or with confidence lower than 0.75 (on a scale of 0–1), as well as participants that were not tracked successfully (with <90% of all frames recognized).

OpenFace provides gaze angle values for each frame, which are provided in *x* and *y* coordinates with the camera as the reference point. To account for participants’ height, all values were centered around the median. We calculated the mean of the absolute values (this equals the mean deviation of the median of the gaze), and the means for the first derivate (speed of the gaze) and the second derivate (acceleration of gaze) for the whole conversation. Further, we measured the participant’s mimicking of the actress’ facial gaze using correlations within 10 s timeframes.

We analyzed the audio recording of each participant’s voice using the python library Librosa^74^ and Parselmouth Praat Scripts in Python by David Feinberg^75^ to extract the following prosodic features for each frame: *f*0 (fundamental frequency of vocal oscillation), jitter (pitch perturbations), and shimmer (amplitude perturbations) and the root-mean-square energy. All of these features are frequently used in voice analysis and have shown to vary in different clinical conditions.^77,78^ To compare the two groups, we calculated the respective means over all frames of the whole conversation for each feature for each subject. For the machine-learning analysis of the diagnostic value of the voice, we additionally extracted with Librosa first 40th Mel-frequency cepstral coefficients, which capture essential information from voice signal and have been used in automatic speech recognition as well as in voice disorder classification.^79^

### Machine-learning analysis

This work builds on and extends a preliminary conference presentation directed at a machine-learning audience.^41^ In addition to the results of Drimalla et al.,^41^ this paper compares the machine-learning approach to ratings of clinical experts, explores differences in facial features, voice features, and gaze features between subject groups. To investigate the predictive value of the features from each of the three domains (facial expression, gaze, and voice), we used machine-learning methods. For machine-learning analysis, the library scikit-learn (https://scikit-learn.org) for Python was used.

First, to gain a better representation for the machine-learning analysis, we extracted secondary features. For facial AUs and gaze angles, we calculated mean, standard deviation, minima, maxima, time points of maximum, skewness, and kurtosis. The calculations were done separately for each of the seven parts of the conversation. As a result, we used 715 features for the facial expression and 85 features for the gaze behavior. For voice analysis, we calculated the first forty mel frequency cepstral coefficients (MFCC), energy, the mean and standard deviation of the fundamental frequency and different measures of shimmer and jitter. Due to computational power, we limited us to these 58 primary features. Gender was included as a feature in all models to ensure that the model could select certain features as being more predictive for certain genders.

For each participant, we built only one feature vector that contains the aggregate statistical measures across all video segments. Thus, the data for the machine-learning analysis did not include any nested repeated measures. Using this feature representation, we conducted a machine-learning analysis to explore the diagnostic value of non-verbal social behaviors (gaze/face/voice), i.e., the prediction of whether a participant had received a diagnosis of ASD. We built models to map the feature vectors to a value of a binary (ASD vs. NT) decision function involving balanced classes.

We compared different machine-learning approaches to solve this problem in a previous paper.^41^ In this paper, we used a random forest approach,^80^ which classifies the participants averaging the results of an ensemble of 1000 different decision trees on different subsets of data and input variables. The maximum depth of the trees and the minimum number of samples per leaf were the hyperparameters: For depth of trees the used values were [1, 2, 4, 8, 16, 32, 64], and for samples per leaf [1, 2, 4, 8, 16, 32, 64].

To train and test the machine-learning models, we used a leave-one-out cross-validation. To calculate the AUC, we trained separately for each participant a model with the data of the other participants and then tested each model only on the respective left-out participant. No data of the test-participant was used for training the model. Inside the training data, we tuned the hyperparameter with a nested three-fold-cross-validation.

### Analysis of the data by clinical experts

Eight psychologists/psychiatrists with ample experience in diagnosing and treating individuals with ASD evaluated 10 randomly selected videos of different participants each, which were unknown to them. To match the rating task as closely as possible to the machine-learning task, experts were asked to focus on non-verbal behavior only (facial expressions, voice characteristics, and gaze behavior). After watching a video, they were instructed to rate on a visual analog scale where they would locate the participant on the autism spectrum, i.e., to estimate the amount of autistic traits/symptomatology that the individual presents with. The scale ranged from “neurotypical” to “ASD” with a vertical line in the middle that indicated the point at which autism symptomatology reaches clinical significance and warrants a diagnosis of ASD. We assigned the videos randomly to the raters with no predefined proportion of individuals with and without ASD. Each video was rated once.

### Reporting summary

Further information on research design is available in the Nature Research Reporting Summary linked to this article.

## Supplementary information

Supplementary Information

Reporting Summary

## Data Availability

The datasets generated and analyzed during the current study are not publicly available due to privacy restrictions of the video data but are available from the corresponding author on reasonable request.

## Code availability

The SIT is available from the corresponding author on reasonable request. From 2024 on, the SIT is publicly available online via https://edoc.hu-berlin.de/handle/18452/21028 under CC BY-NC-ND 4.0 license. The code used for analyzing the data is publicly available on Github: https://github.com/drimalla/ml-autism.
