# Author Correction: Towards the automatic detection of social biomarkers in autism spectrum disorder: introducing the simulated interaction task (SIT)

**DOI:** 10.1038/s41746-022-00563-3

**Published:** 2022-02-10

**Authors:** Hanna Drimalla, Tobias Scheffer, Niels Landwehr, Irina Baskow, Stefan Roepke, Behnoush Behnia, Isabel Dziobek

**Affiliations:** 1 Department of Psychology, Humboldt-Universität zu Berlin, Unter den Linden 6, 10099 Berlin, Germany; 2 Berlin School of Mind and Brain, Humboldt-Universität zu Berlin, Unter den Linden 6, 10099 Berlin, Germany; 3 Digital Health Center, Hasso Plattner Institute, University of Potsdam, Prof.-Dr.-Helmert-Str. 2-3, 14482 Potsdam, Germany; 4 Institute of Computer Science, University of Potsdam, Am Neuen Palais 10, 14469 Potsdam, Germany; 5 Leibniz Institute for Agricultural Engineering and Bioeconomy, Max-Eyth-Allee 100, 14469 Potsdam, Germany; 6 Department of Psychiatry, Charité-Universitätsmedizin Berlin, Campus Benjamin Franklin, Hindenburgdamm 30, 12203 Berlin, Germany

**Keywords:** Diagnosis, Signs and symptoms, Autism spectrum disorders

Correction to: *npj Digital Medicine* 10.1038/s41746-020-0227-5, published online 28 February 2020

The original version of the published Article included a power calculation that was unrelated to the main analysis performed in this study. The second sentence of the second paragraph of the methods section referred to this power analysis. To improve clarity and reproducibility, the sentence has been removed from the methods. Additionally, the original version of the Supplementary Information contained typographical errors in the Supplementary Tables, which have been corrected. The HTML and PDF versions of the Article have been corrected.

## Supplementary information

Supplementary Material

