# Supplementary material for: Towards the automatic detection of social biomarkers in autism spectrum disorder: introducing the simulated interaction task (SIT)
Source: NPJ Digit Med. 2020 Feb 28;3:25. doi: 10.1038/s41746-020-0227-5 (PMC7048784; doi:10.1038/s41746-020-0227-5)
Supplement: Supplementary file 1 — Supplementary Information [file 41746_2020_227_MOESM1_ESM.pdf]

SUPPLEMENTARY MATERIAL

Hanna Drimalla, Tobias Scheffer, Niels Landwehr,
Irina Baskow, Stefan Roepke, Behnoush Behnia, Isabel Dziobek

FACIAL EMG PREPARATORY STUDY

Purpose of the Study

This study aimed to validate the SIT as a task evoking non-verbal social communication behavior. Using EMG's high-precision measurement of facial muscle activity, we selected the features of interest and hypothesized patterns of non-verbal behavior for the ASD study.

Participants

Forty healthy men participated in the study. Exclusion criteria were a prior history of psychological, neurological or psychiatric disorders, facial sensitivity disorders, paralysis, and severe acne. After pre-analysis, we excluded five participants, three due to technical reason and two due too many movement artefacts, and analyzed data of 35 participants (age range: 18 to 36 years, mean age: 26 years).

Procedure

The study took place in a quiet laboratory with constant lighting conditions. After having given informed consent, participants' autistic traits were assessed via the Autism-Spectrum Quotient[1].

Before attaching the EMG electrodes, the participants' facial skin was cleaned with an alcohol pad. Bipolar surface electrodes were then attached over the Zygomaticus Major, Corrugator Supercilii and Levator Labii muscles on the non-dominant side of the face, following suggested procedures by Fridlund and Cacioppo[2]

Participants were asked to sit in front of a computer screen and were given instructions, after which the experimenter left the room and the participant started the SIT. While undergoing the task, facial EMG was recorded. Before each part of the conversation, a fixation cross was shown for 1500ms as a baseline period.

After the simulated conversation, the participants were asked whether they had noticed anything special or unusual about the experiment. Further, participants were asked whether they have behaved as they would in a real interaction — on a Likert scale from 1 [not at all] to 5 [very much].

The study was approved by the ethics committee of the Humboldt-Universität zu Berlin and was conducted in accordance with the Declaration of Helsinki.

EMG-Recording

Electrode impedance was kept below 50kΩ. The EMG signals were amplified with EMG amplifiers (Becker Meditec, Karlsruhe, Germany; gain =1230; band pass 19Hz - 500Hz). The raw EMG signals were sampled at 1000Hz and digitized with 16bit resolution. Signals were RMS (root mean square) integrated with a time constant of 50ms and down sampled to 20Hz.

Facial muscle activity was z-standardized within muscles and averaged in intervals of 500ms. To check for artefacts, the video recordings of all participants were screened visually. Additionally, an automatic outlier detection was performed excluding all values from a baseline-corrected trial that were three standard deviations above the mean value of all trials.

For the analysis of facial expression, we focused on the muscles that have been shown to be relevant for the emotions joy (positive food experience) and disgust (negative food experience). Joy is expressed in the face mainly through the activity of the muscle Zygomaticus Major, which raises the corner of the mouth. For the analysis of disgust, we

focused on relevant muscles and AUs, respectively, i.e., Levator Labii, which lifts the lower part of the lip, and the Corrugator Supercilii, which is active in frowning and associated with emotions of negative valence in general as well as with disgust in particular.

As speech movements may disturb the EMG signal, we analyzed only parts of the conversation in which the participants were not talking. We compared the activity of the three muscles in the neutral part (baseline) with their activity in the positive and negative part.

Results

The activity of the three muscles differentiated between the distinct parts of the conversation (see Table 3 of the main manuscript and Supplementary Figure 1), as evident in a significant interaction effect between muscles and parts ($F(4, 27) = 6.79$, $p < 0.0001$). Post hoc Wilcoxon-Tests (p-level corrected for six comparisons) revealed that the participants' mean activity of Zygomaticus Major was significantly higher during both emotional parts of the conversation (positive: $Mdn = -0.20$; $Z = 111$, $p < 0.001$, negative: $Mdn = -0.16$, $Z = 73$, $p < 0.0001$) than in the neutral part ($Mdn = -0.29$). In contrast, the activity of Levator Labii was higher in the negative ($Mdn = -0.12$) than neutral part ($Mdn=-0.24$), $Z=147$, $p = 0.006$.

As they are representing expressions of positive and negative valence, we analyzed the activity of Corrugator and Zygomaticus in relation to an individual's autistic traits. A linear mixed model revealed an interaction between autistic traits and the two muscles on their activity ($b = -0.05$, $SE = 0.02$, $z = -2.21$, $p = .03$), such that a higher Corrugator's and a lower Zygomaticus' activity was associated with more pronounced autistic traits. The participants stated that they behaved similar to a real conversation on a medium level ($M=2.97$, $SD=1.13$).

Supplementary References

1. Baron-Cohen, S., Wheelwright, S., Skinner, R., Martin, J. & Clubley, E. The Autism-Spectrum Quotient (AQ): Evidence from Asperger Syndrome/High-Functioning Autism, Malesand Females, Scientists and Mathematicians. Journal of autism and developmental disorders 31, 5–17; 10.1023/A:1005653411471 (2001).

2. Fridlund, A. J. & Cacioppo, J. T. Guidelines for Human Electromyographic Research. Psychophysiology 23, 567–589; 10.1111/j.1469-8986.1986.tb00676.x (1986).

SUPPLEMENTARY TABLES

Supplementary Table 1. Confusion matrix of face classifier.

		Actual Class	
		HC	ASD
Predicted Class	HC	31	14
	ASD	12	22

Supplementary Table 2. Confusion matrix of gaze classifier.

**Gaze**		Actual Class	
		HC	ASD
Predicted Class	HC	26	17
	ASD	17	19

Supplementary Table 3. Confusion matrix of voice classifier.

**Voice**		Actual Class	
		HC	ASD
Predicted Class	HC	33	10
	ASD	10	26

Supplementary Table 4. Confusion matrix of total classifier.

**Total**		Actual Class	
		HC	ASD
Predicted Class	HC	34	12
	ASD	9	24

Supplementary Table 5. Confusion matrix of experts.

**Experts**		Actual Class	
		HC	ASD
Predicted Class	HC	37	16
	ASD	6	20

SUPPLEMENTARY FIGURES

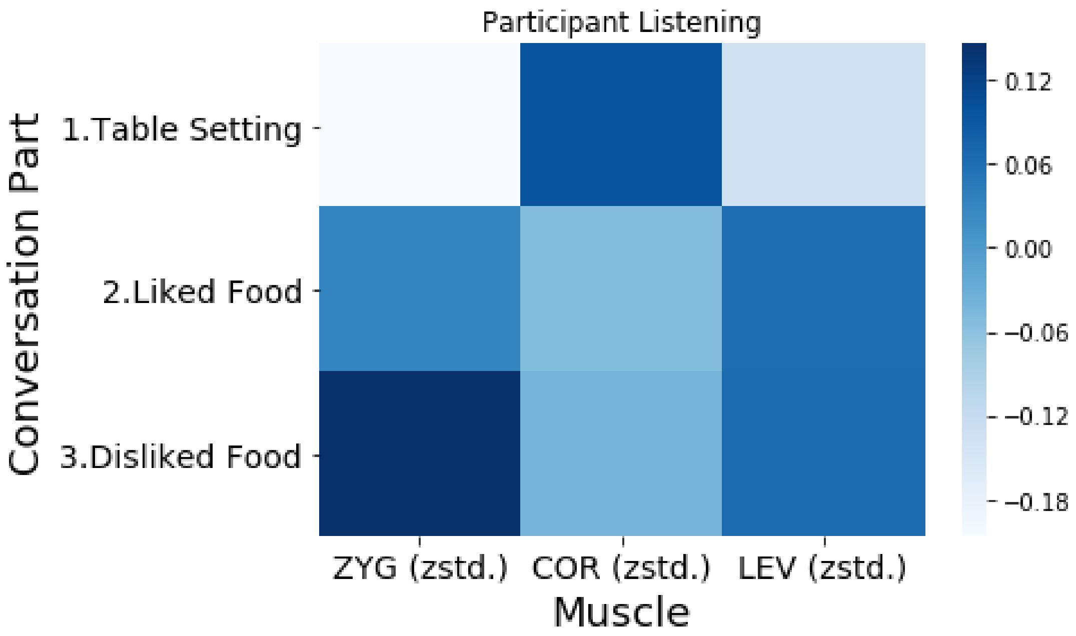

Supplementary Figure 1. Mean standardized activity in each conversation part of each muscle, Zygomaticus Major (ZYG), Corrugator Supercilii (COR) and Levator Labii (LEV).

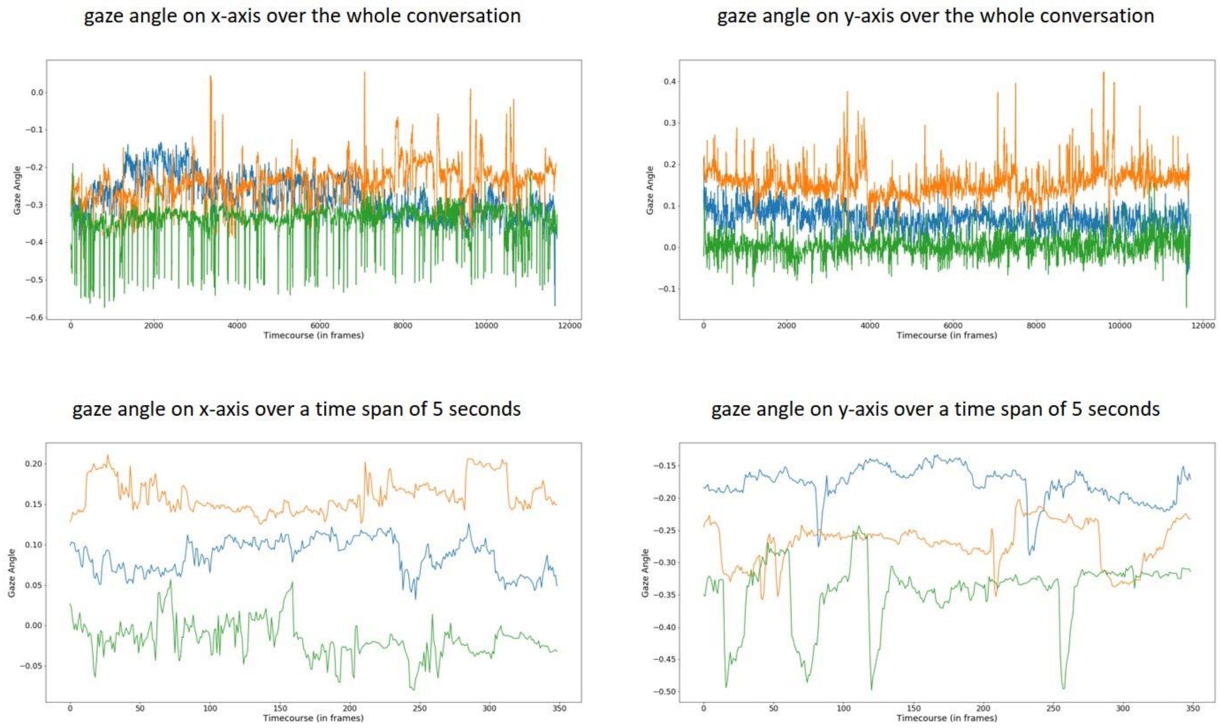

Supplementary Figure 2. Gaze angle estimation by OpenFace of three sample participants for both x- and y-angle. The first row shows the complete conversation (recorded with 30frames per second), the second row shows five seconds during the conversation.
